# Association between pesticide exposure and paraoxonase-1 (*PON1*) polymorphisms, and neurobehavioural outcomes in children: a systematic review

**DOI:** 10.1186/s13643-020-01330-9

**Published:** 2020-05-09

**Authors:** Nkosinathi Banhela, Pragalathan Naidoo, Saloshni Naidoo

**Affiliations:** 1 Discipline of Public Health Medicine, School of Nursing and Public Health, College of Health Sciences, University of KwaZulu-Natal, Howard College Campus, Durban, 4000 South Africa; 2 Discipline of Medical Biochemistry and Chemical Pathology, School of Laboratory Medicine and Medical Science, University of KwaZulu-Natal, Howard College Campus, Durban, 4000 South Africa

**Keywords:** Pesticide exposure, Neurodevelopment, Neurobehavioural outcome, Paraoxonase-1, Single nucleotide polymorphism, Genotype, Organophosphate

## Abstract

Environmental factors such as pollution, pesticide exposure and socio-demographic location have been implicated as a pressure capable of altering genetic make-up. Altered genetic sequence of genes encoding enzymes may result in single nucleotide polymorphism (SNP). Of peculiar interest is the genetic variance on the paraoxonase-1 gene induced by pre- and postnatal exposure to pesticides. SNP have been reported on the paraoxonase-1 gene and post-xenobiotic exposure and are presumed to alter gene sequence and ultimately enzymatic activity. The altered enzymatic activity may facilitate neurodevelopment disorders. Autism spectrum disorders (ASD) and attention deficit hyperactivity disorder (ADHD) are among the neurodevelopment disorders of which prevalence is concurrently associated with increasing environmental xenobiotic exposure. The variance on xenobiotic metabolising genes is associated with altered neurodevelopment outcome and ultimately altered neurobehavioural outcome. Prime interests of this systematic review were to establish an understanding of the sequences on the paraoxonase-1 gene associated with adverse neurobehavioural outcome. An in-depth literature search was conducted using the term combination “pesticide exposure, pre- and postnatal exposure, organophosphates/organophosphorus, single nucleotide polymorphism, paraoxonase-1 (PON-1), neurodevelopment/neurobehavioural outcome in child/infant”. Articles published from the year 2000 to 2018 were considered for review. The result showed that variance on the PON1-108 and 192 alleles could be implicated in the development of altered neurobehavioural outcomes.

## Background

Organophosphates (OP) are a group of insecticide pesticides which includes chlorpyrifos, parathion, diazinon and fenthion. Organophosphates mode of action is by inhibiting the acetylcholinesterase enzyme, preventing the breakdown and uptake of acetylcholine [1]. This then leads to an accumulation of acetylcholine and sustained excitation of neurons, which ends up killing the insect. In humans, however, the health outcome post exposure can be acute or chronic depending on the duration of exposure. The sustained excitation of neurons in humans may result in symptoms such as salivation, nausea, vomiting, lacrimation and seizures [1]. Whilst chronic exposure to organophosphates may induce adverse respiratory [2], neurotoxic [1, 3] and neurodevelopmental outcomes [4, 5].

Globally, there is a significant prevalence of neurodevelopmental disorders in children such as autism spectrum disorders (ASD), epilepsy and attention deficit hyperactivity disorder (ADHD) [6–8]. Requena et al. reported the prevalence of epilepsy to be significantly associated with high pesticide use, among the South-East Spain population [9]. In 2015, Baxter et al. performed a systematic review on the global burden of ASD and reported a point prevalence of 7.6 cases per 1000 population in 2010 [8]. Kogan et al. reported an ASD prevalence of 2.5% among 3–17 years age groups in the USA for 2010 [10]. In a systematic review focusing on the prevalence of neurodevelopmental disorders in low- and middle-income countries, for every 1000 population, the median pooled prevalence for any neurodevelopment disorders (NDD) was 7.6 (95% CI 7.5–7.7), with 11.3 (95% CI 11.7–12.0) for neurological disorders and 3.2 (95% CI 3.1–3.3) for mental conditions such as attention deficit hyperactivity disorder (ADHD).

Sociodemographic factors [11], genetics [12], adverse gestational outcomes [13] and environmental toxicants [12] have been implicated in the aetiology of neurodevelopmental disorders. The detrimental impact of environmental toxicants such as pesticides on child neurodevelopment has been widely reported [14–16].

In several developing countries, pesticides are being used extensively in agriculture and in vector control [17]. In Southern Africa, pyrethroids and dichlorodiphenyltrichloroethane (DDT) are being extensively used to control the growth of the Anopheles mosquito which is responsible for malaria transmission in multiple countries [17, 18]. Furthermore, there is an unregulated use of pesticides in small-scale agricultural farming in most African countries [17, 18]. The continuous and unregulated use of pesticides results in persistence of pesticides in soil and water and consequently in human and animal bodies [19]. This increases exposure among individuals living in agricultural and malaria endemic settings [20, 21].

Pesticides have been known to induce a selective pressure, capable of altering single nucleotide sequences on genes. Organophosphates are metabolised by the enzymes paraoxonases (POase) and arylesterases (ARYase) which are encoded by the paraoxonase-1 (*PON1*) gene [18, 19]. Paraoxinase-1 is an A-esterase enzyme found in the liver and plasma, facilitating the breakdown of oxons of OP (paraoxon) [4]. Single nucleotide polymorphisms (SNP) within xenobiotic (pesticide) metabolising genes can negatively influence the biotransformation and detoxification of pesticides [22]. Polymorphic status of the PON1 gene for an individual may be involved in the determination of OP susceptibility among exposed individuals and may determine health outcome [3–5, 21, 23]. Two polymorphisms have been identified on the coding region of PON1 which are Q192R and L55M and five identified on the promoter region, which are -108(C/T), -126(G/C), -162(A/G), -832 (G/A) and -909(C/G) [4]. Polymorphisms on the *PON1* gene can negatively alter the metabolising ability of the POase and ARYase enzymes [3].

Developmental neurotoxicity induced by exposure to pesticides is a public health concern because of its associated damaging effects on the central nervous system (CNS), resulting in a decline in cognitive ability [24]. SNP on the PON1 gene can negatively impact neurodevelopment, as acetylcholine is a potent neurotransmitter for the development and functioning of the CNS [21, 25]. Maternal exposure to pesticides during pregnancy can negatively impact the growth and development of the foetus in utero, leading to adverse birth outcomes [7] and neurodevelopment [16] as the neonates progress in age.

Genetic variability on the PON1 gene induced by exposure to OP has been reported [4, 23]; however, distinctive associations with neurobehavioural health outcomes in children are still due [3, 5, 21].

Thus, the aim of this systematic literature review was to gather information on the associations between pesticide exposure (organophosphates), single nucleotide polymorphisms in the *PON1* gene, and further associate with neurobehavioural health outcomes in children.

## Methods

This systematic review gathered relevant information from the literature about pesticide exposure in pregnant women, single nucleotide polymorphisms in the *PON1* gene and neurobehavioural health outcomes in their offspring. A narrative approach was undertaken to review available data for this study.

### Search strategy

Articles considered for the review were published between the years 2000 and 2018. Articles were retrieved using online search engines and library sources, including the Institute for Scientific Information (ISI) Web of Knowledge, Cochrane library, Google, Google Scholar, PubMed Search and Science Direct. The key terms used to generate the search were “pesticide exposure” and “prenatal exposure”, “organophosphates/organophosphorus” and “single nucleotide polymorphisms”, “paraoxonase-1 (PON1)”, and “neurodevelopment/neurobehavioural outcome in child/infant”. Individual terms and a combination of terms were used to search for articles. Unpublished studies and the grey literature were not reviewed for this search. Reference lists of retrieved articles were also reviewed, and relevant articles were retrieved.

### Criteria used for selecting studies

Inclusion criteria for selected articles were (a) articles must be written in English, (b) articles must give details of maternal pesticide exposure and (c) articles must document the presence of single nucleotide polymorphisms in the *PON1* gene and (d) neurobehavioural health outcomes in children. Children were defined as newborns to 16 years of age. Study designs which were of interest in this review could either be of a prospective cohort, longitudinal cohorts and case controlled studies.

Exclusion criteria were (a) articles published prior to the year 2000, (b) articles in languages other than English were excluded, (c) articles including pesticides other than organophosphates were excluded, (d) if the evidence of pesticide exposure was unclear or not documented the article was excluded, (e) articles reporting on SNPs in genes other than the *PON1* gene were excluded and (f) non-neurobehavioural health outcomes were excluded from the search (Table 1).

**Table 1:** Inclusion and exclusion criteria

Inclusion	Exclusion
Year published, 2000–2018	< 1999
Pesticide of interest: Organophosphate/organophosphorus and metabolite	Other pesticide like DDT, carbamates and pyrethroids
Pre- and postnatal exposure to pesticide well defined	Exposure to pesticide not well defined/exposure to another xenobiotic beside OP
SNP of interest on the PON1 gene	Other SNP besides those on PON1 gene
Health outcome: Neurobehavioural health outcome (cognitive, behavioural, sensory, motor and morphology)	Other health outcome beside neurobehavioural

*DDT* dichlorodiphenyltrichloroethane, *SNP* single-nucleotide polymorphism, *OP* organophosphate, *PON1* paraoxonase-1

### Study selection process

**Fig. 1:**
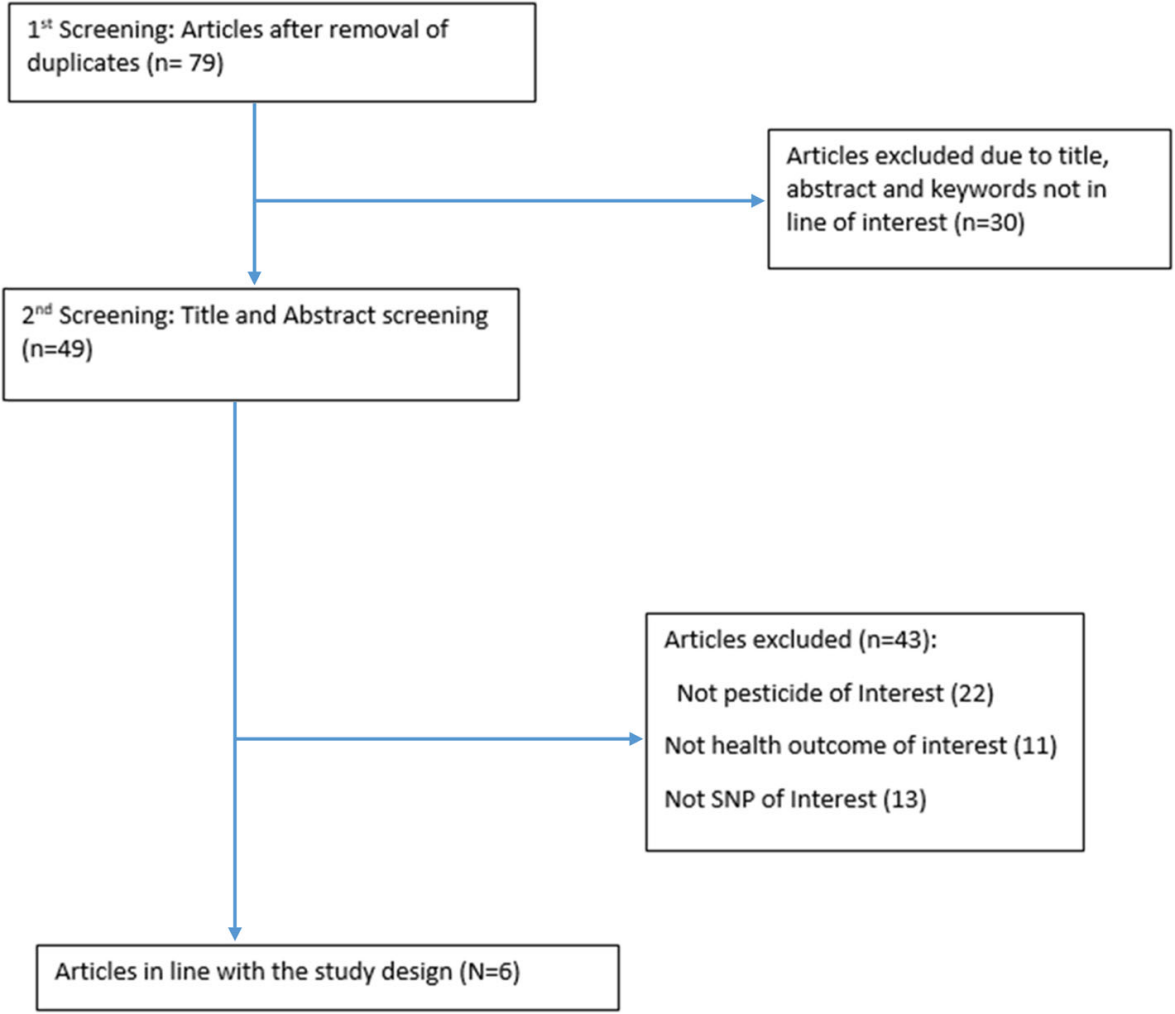
Prisma flow diagram presenting the article selection process for this review


The literature search strategy was developed by Nkosinathi Banhela (NB) who is the first author on this paper and was approved by his PhD supervisor, Dr Saloshni Naidoo (SN) a co-author on this paper. Articles were screened for inclusion eligibility in this study by NB and confirmed by PN. Two reviewers (NB and PN) reviewed the title, abstracts and full-text for eligibility of the studies to be included in the current study, and a third reviewer checked for discrepancies (SN). The online search identified 179 relevant citations. The titles and abstracts of 76 articles were reviewed in detail; 103 articles were reviewed not to be in line with the inclusion criteria. After a thorough review of the titles and abstracts of articles, the full-texts of 49 articles were retrieved and screened; 27 articles were not in congruency with the inclusion criteria. Six articles met the inclusion criteria. Figure 1 summarises the online search using the PRISMA flow diagram. The reference manager tool that was used to record and manage the referencing and the reference list is “Endnote X9” referencing managing software (Table 2).

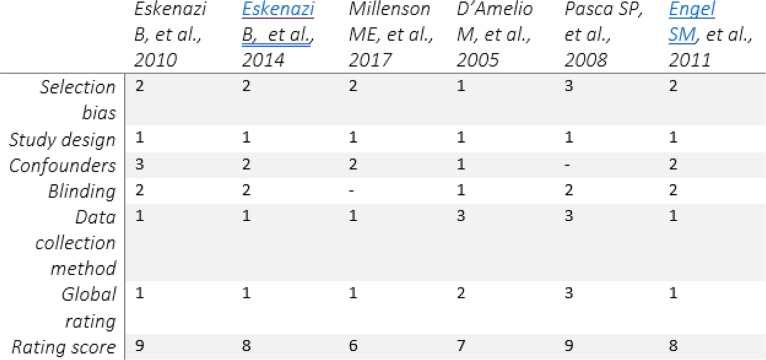

**Table 2:**
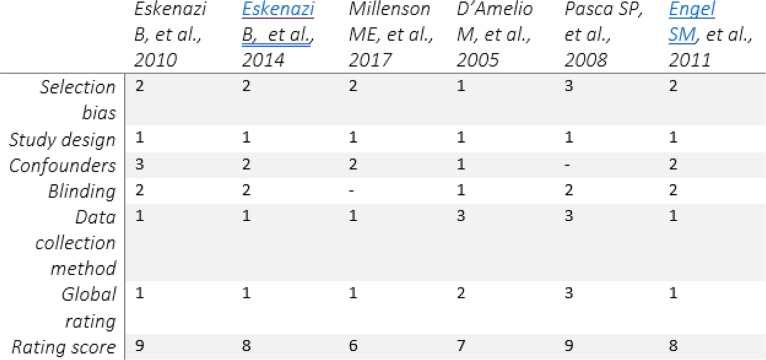
EPHPP quality assessment tool

### Data extraction and presentation

Data from the selected articles were extracted into an Excel spreadsheet. The Excel spreadsheet was structured to visualise alignment of selected articles with the inclusion and exclusion criteria. NB performed the initial synthesis of collected data; PN verified and checked the reported findings on data. Data were extracted relating to the study title, authors, publication year, sample size, study design, biological sample analysed, neurobehavioural tests used, age of children assessed and outcomes of neurobehavioural tests (see Table 3).

**Table 3:** Representing characteristics of articles chosen for inclusion for current study

Title	Authors	Sample size	Study design	Pesticide/metabolites and screening tool used	Genotype(s)	Neurodevelopment tool and age	Association and stat	Conclusion
PON1 and neurodevelopment in children from the CHAMACOS study exposed to organophosphate pesticides in utero.	Eskenazi et al. [5]	*n* = 353 (children of participants of the CHAMACOS study)	Longitudinal birth cohort	Dialkyphosphate and metabolites. GCMS	PON1 (-108T) PON1 (192 QQ)	Mental Development Index (MDI) Bayley Scale of Infant Development (BSID) Psychomotor Development Index (PDI) Age 2 year olds	PON1 (-108) = − 3.2 (− 9.8–3.5), *p* = 0.98 (MDI) = − 2.3 (− 7.8–3.3), *p* = 0.91 (PDI) PON1 (-192) = − 6.5 (− 15.6–2.6), *p* = 0.33 (MDI) = − 1.7 (− 8.7–5.4), *p* = 0.53 (PDI)	*PON1*_*−108T*_ related to MDI and, to a lesser extent, PDI in toddlers. Adds to the growing evidence that the *PON1* gene is associated with an array of neurologic end points in adults and in children
Organophosphate pesticide exposure, PON1 and neurodevelopment in school-age children from the CHAMACOS study	Eskenazi et al. [21]	[K-CPT] (*n* = 296) {WISC-IV]-(*n* = 327)	Longitudinal birth cohort	DAP and metabolites. GCMS Enzymatic activity of ARYse and POase. Spectrophotometer	PON1 (-108T) PON1 (-192Q)	Conners’ Kiddie Continuous Performance Test (K-CPT) at 5 years old and the Wechsler Intelligence Scale for Children (WISC-IV) at 7 years old	WISC positively associated with ARYase, 95% CI = 1.6, 6.4 *PON1*_*−108*_ weakly modified DAPS and K-CPT scores (*p* = 0.21) and WISC verbal IQ (*p* = 0.71) DAPs and IQ strongest for children of mothers with lowest-tertile ARYase levels (*p* = 0.27)	PON1 enzyme levels during pregnancy may also increase susceptibility of children to neurotoxicity from OP pesticide exposure
Urinary organophosphate insecticide metabolite concentrations during pregnancy and children’s interpersonal, communication, repetitive and stereotypic behaviours at 8 years of age. The home study	Millenson et al. [26]	*W* = 224 mothers (PON1R192Q: *n* = 531, PON1L55*M*: *n* = 458) and children (PON1R192Q: *n* = 532, PON1L55*M*: *n* = 478)	Birth cohort	OP and metabolites. Samples analysed by CDC	PON1 (R192Q) PON1 (L55M)	Conners’ Parent Rating Scales-Revised (CRS-R), Conners’ Continuous Performance Test (CPT) Behaviour Assessment System for Children-2 (BASC2) Age 8 years	PON1−108TT genotype, ΣDAP concentrations were associated with 2.5-point higher (95% CI − 4.9, 9.8) SRS scores; however, the association was not different from the 1.8-point decrease (95% CI − 5.8, 2.2) among children with PON1−108CT/CC genotypes (ΣDAP × PON1−108 *p* value = 0.54). The association between ΣDAP concentrations and SRS scores was not modified by PON1192, *p* = 0.89	Maternal PON1192QQ associated with PON155MM and parent reported ADHD-LP in children Maternal genotype significantly associated with ADHD-LP
Paraoxonase gene variants are associated with autism in North America, but not in Italy: possible regional specificity in gene–environment interactions	D’Amelio et al. [27]	177 Italian and 107 Caucasian-American	Case control study	OP diazinone. HPLC	PON1 C 108T, L55M and Q192R	ASD-diagnosis, method not specified Age: population based/not specified	(Q192R: v2 ¼ 6.33, 1 df, *p* = 0.025), transmission/disequilibrium tests (Q192R: TDT = 5.26, 1 df, *p* = 0.025), family based association tests (Q192R and L55M: FBAT *Z* = 2.291 and 2.435 respectively, *p* = 0.025) and haplotype-based association tests (L55/R192: HBAT *Z* = 2.430, *p* = 0.025)	Caucasian-American and not Italian families display a significant association between autism and PON1 variants, OP exposure could be implicated in Autism
Paraoxinase 1 activities and polymorphisms in autism spectrum disorders	Pasca et al. [28]	*n* = 50 ASD and 30 control	Case control study	No pesticide mentioned	PON1 (Q192R) and PON1 (L55M)	Diagnostic and statistical manual of mental disorders, fourth edition revised (DSM-IVR) Age 6–7 years old	PON1 arylesterase and PON1 paraoxonase activities were decreased in autistic patients (respectively, *p* < 0.001, *p* < 0.05), no association between genotype and autism distribution	Bioavailability and the catalytic activity of PON1 are impaired in ASD
**Prenatal exposure to organophosphates, paraoxonase 1 and cognitive development in childhood**	Engel et al. [29]	Mothers (*n* = 360) Children 1 year (*n* = 200), 2 years (*n* = 276) and 6–9 (*n* = 169) years of age	Prospective Multiethnic cohort	Organophosphate and metabolites. GCMS	PON1 (Q192R)	The Bayley Scales of Infant Development, 2nd edition (BSID-II) Age 12 moths, 24 months and 6–9 year olds	ΣDAP and ΣDMP tertials of exposure were associated with a decrease in the MDI [log10 ΣDAP, *β* = − 3.29; 95% confidence interval (CI), − 5.88 to − 0.70]. ΣDAP metabolite level was inversely associated with the 24-month MDI (*β* = − 2.08; 95% CI, − 4.60 to 0.44) in multivariate adjusted models PON1 192 QR/RR genotype experienced approximately a 5-point decline on the MDI with each log10 unit increase in ΣDAP or ΣDMP	Exposure to organophosphates is negatively associated with cognitive development, particularly perceptual reasoning, with evidence of effects beginning at 12 months and continuing through early childhood

*ASD* autism spectrum disorders, *ADHD* attention deficit hyperactive disorder, *DAP* dialkyphosphate, *DMP* dimethyl phosphate, *BSID-II* Bayley Scale of Infant Development, *MDI* Mental Development Index, *PDI* Psychomotor Development Index, *PON1* paraoxonase-1, *WISC* Weschler Intelligence Scale for Children

### Data synthesis and analysis

Data from articles selected to be included were tabulated to represent study sample size, study design, screening tool used to measure pesticide of interest, genotype of interest, neurodevelopmental tool used and age it was administered. The above-mentioned parameter was then compared among the articles selected for inclusion in the study. Further, the PON1 genotype was associated with the observed/screened neurobehavioural outcome and the statistical score used for the associations (see Table 4). Neurobehavioural outcomes explored in each selected article were characterised to have either cognitive, behavioural, sensory, motor and morphological effect. A meta-analysis for the current available data could not be performed as there were no randomised controlled trials found in the literature. While the health outcomes under study were in the same category of neurodevelopmental outcomes, different measuring instruments were used by the studies. The neurobehavioural tests in the different papers rendered results which were either behavioural, cognitive, sensory, motor or morphological, making it difficult to compare for a meta-analysis.

**Table 4:** Representing PON1 genotype and associated health outcome

Title	Authors	Outcome		Conclusion
PON1 and neurodevelopment in children from the CHAMACOS study exposed to organophosphate pesticides in utero	Eskenazi et al. [5]	**Bayley MDI*β*(95% CI)** PON1-_108_ CC, reference (*p* < 0.01) CT, − 3.9 (− 6.6 to − 1.2) TT, − 5.7 (− 9.0 to − 2.5) PON1-_192_ RR, reference (*p* = 0.65) QR, − 0.5 (− 3.4 to − 2.4) QQ, 0.7 (− 2.6 to 4.0) **Bayley PDI** PON1-_108_ CC, reference (*p*=0.07) CT, − 1.4 (− 3.8 to − 1.0) TT, (− 5.7 to 0.2) PON1-_192_ RR, reference (*p* = 0.10) QR, 0.3 (− 2.2 to 2.9) (*p* = 1.0) QQ, 2.4 (− 0.5 to 5.4) (*p* = 1.0) **CBCL PDD** PON1-_108_ CC, reference (*p* = 0.14) CT, 1.5 (0.7 to 3.3) TT, 2.0 (0.8 to 5.1) PON1-_192_ RR, reference (*p* = 0.94) QR, (0.4 to 2.2) QQ, (0.4 to 2.4)		The PON1-108T allele in children associated with poorer Bayley MDI scores and with somewhat poor PDI scores.
Organophosphate pesticide exposure, PON1, and neurodevelopment in school-age children from the CHAMACOS study	Eskenazi et al. [21]	**Mother** PON1-108 **KCPT ADHD [*β*(95%)] (at age 5), WISC PRI [*β*(95%)](age 7**) CC, reference (*p* = 0.41), reference (*p* = 0.69) CT, 2.9 (− 1.9 to 7.8), − 2.6 (− 6.8 to 1.6) TT, 2.1 (− 3.6 to 7.9), − 2.8 (− 7.0 to 1.5) PON1 192 **KCPT ADHD [*β*(95%)] (at age 5), WISC PRI [*β*(95%)](age 7)** RR, reference (*p* = 0.86), reference (*p* = 0.65) QR, 2.1 (− 2.9 to 7.1), − 2.6 (− 7.0 to 1.7) QQ, − 0.5 (− 6.1 to 5.1), 1.0 (− 3.7 to 5.8) **Child** PON1 108 **KCPT ADHD [*β*(95%)] (at age 5), WISC PRI [*β*(95%)](age 7)** CC, reference (*p* = 0.88), reference (*p* = 0.52) CT, − 0.2 (− 4.9 to 4.6), − 0.5 (− 4.6 to 3.6) TT, 0.6 (− 5.4 to 6.5), − 1.8 (− 7.0 to 3.4) PON1 192 **KCPT ADHD [*β*(95%)] (at age 5), WISC PRI [*β*(95%)](age 7**) RR, reference (*p* = 0.96), reference (*p* = 0.21) QR, 5.0 (0.1 to 9.9), − 4.6 (− 9.0 to − 0.2) QQ, 0.1 (− 5.8 to 5.9), − 3.4 (− 8.5 to 1.7)		Maternal and child PON1 genotype was not related to performance on K-CPT or WISC; WISC scores were lowest in children and children of mothers who carried the PON1 108TT genotype. Maternal PON1 108 weakly modified the relationship of maternal DAPS and K-CPT score and WISC verbal IQ. PON1 genotype and enzyme levels may be related to performance on certain domains of neurodevelopment in school age children.
Urinary organophosphate insecticide metabolite concentrations during pregnancy and children's interpersonal, communication, repetitive, and stereotypic behaviours at 8 years of age: The home study	Millenson et al. [26]	PON1 108TT associated with [∑DAP] = *β* 2.5 point higher (95% CI − 4.9 to 9.8) PON1 108CT/CC associated with [∑DAP] = *β* 1.8 point decrease (95% CI − 5.8 to 2.2) (*p* = 0.54) PON1 192 modification by ∑DAP = not significantly different *p* = 0.89		PON1 genotype did not modify association between DAP conc. And children social behaviour.
Paraoxonase gene variants are associated with autism in North America, but not in Italy: possible regional specificity in gene–environment interactions	D’Amelio et al. [27]	**Caucasian-American PON1 108*Z*score,*p*value** TC, − 0.310, 0.7566 TT, 0.310 PON1 L55M ML, 2.435, 0.01489 MM, − 2.435 PON1 Q192R RQ, − 2.291, 0.02199 RR 2.291 Italian PON1 108 *Z* score, *p* value TC, 0.290, 0.772 TT, − 0.290 PON1 L55M ML, 0.079, 0.937 MM, − 0.079 PON1 Q192R RQ, 0.000, 1.000 RR, 0.000		Caucasian-American, but not Italian, patients diagnosed with autism were more likely to carry the PON 1-108T allele and not the PON 192R allele, although not significant.
Paraoxinase 1 activities and polymorphisms in autism spectrum disorders	Pasca et al. [28]	**PON1 Q192R [ASD(50), Control (30)],**_**X**_**2,*p*** **QQ,** 26 (52.0%), 43 (50.6%); 0.02, 0.98 QR, 21 (42.0%), 37 (43.5%) RR, 3 (6%), 5 (5.9%) Q/R, 0.73/0.27, 0.72/0.28; 0.01, 0.90 **PON1 L55M** LL, 15 (30.0%), 31 (36.5%); 1.13, 0.56 LM, 30 (60.0%), 43 (50.6%) MM, 5 (10.0%), 11 (12.9%) L/M, 0.60/0.40; 0.62/0.38; 0.08, 0.77		PON1 192 or PON1 55 allelic frequencies not significantly associated with enzymatic levels in ASD and non ASD control PON 1 Q192Q associated with POase activity PON1 enzyme activities are significantly decreased in ASD patients compared to healthy control, irrespective of PON1 polymorphism distribution.
**Prenatal exposure to organophosphates, paraoxonase 1, and cognitive development in childhood**	Engel et al. [29]	**1-year BSID-II Black/Hispanic** PON1 192 (log10 *β* 95% CI) **QQ**, ∑DAP 5.72 (− 0.48 to 11.92), ∑DEP 3.69 (− 0.97 to 8.36), ∑DMP 2.76 (− 2.44 to 7.97) **QR/RR**, ∑DAP − 4.95 (− 7.81 to − 2.07) *p*<0.01, ∑DEP − 1.95 (− 5.36 to 1.47) *p* = 0.06, ∑DMP − 4.47 (− 7.05 to − 1.89) *p* = 0.02	**2-year BSID-II all population** PON1 192 (log10 *β* 95% CI) **QQ**, ∑DAP − 1.04 (− 6.06 to 3.99), ∑DEP − 0.55 (− 4.79 to 3.70), ∑DMP 0.12 (− 4.17 to 4.42) **QR/RR**, ∑DAP − 1.27 (− 4.40 to 1.84) *p* = 0.98, ∑DEP − 0.15 (− 3.51 to 3.21) *p* = 0.88, ∑DMP − 4.47 (− 3.27 to 2.30) *p* = 0.81	Organophosphate negatively associated with cognitive development, particularly perceptual reasoning, starting at year and up to 9 year olds. Mothers carrying PON1 Q192R QR/RR genotype showed decreased mental development scores.

### Quality appraisal

The “Quality Assessment Tool for Quantitative Studies” developed by the Effective Public Health Practice Project (EPHPP) was used to appraise the quality of articles for inclusion in the review [30]. These guidelines assess the quality of studies based on selection bias, study design, confounders, blinding, data collection tools, withdrawal and drop-outs, intervention integrity and analysis. The first author (NB) performed the quality appraisal for articles that were in-line with our current study; then, the reviewer (PN) performed the quality appraisal according to his judgement for the selected articles. Reviewers then cross-checked if the appraisal had any differences which were settled by the third reviewer (SN) (as she is the author that is most experienced in this study). Studies which showed poor quality (< 4 score) were excluded from the review. Articles with a moderate to a strong rating were included in the study (see Tables 2, 5, and 6).

**Table 5:** Definition of rating scores used to assess the study strength

Parameter	Score		
	3	2	1
Pesticide exposure screening method	Pesticide presence performed by main researcher and tools used to determine load well defined, i.e. GCMS and HPLC	Pesticide load determined by sending samples to a private research company, i.e. lab and CDC	Pesticide exposure done by questionnaire, hospital record etc.
Neurodevelopment assessment tool	BSII, WSID	MDI, PDI	Assessment performed by questionnaire
Presence of SNP on PON1	SNP on the PON1 Q192R and -108TT	SNP on PON1 L55M	SNP on PON1 -126 GC, -162AG, -832GA and -909CG
Study design	Prospective, longitudinal and epidemiological	Case control study	Cross-sectional
Sample size	300	≥ 200	≥ 50
Confounder control	Confounding variables well defined and catered for in the study	Confounding variables are addressed but not all	Confounding variables not defined nor addressed
Funding statement	Funding sources and role of funders in study well stated in the article	Role of funders not explained	Funding sources not mentioned in the article

*BSID* Baylor Scale of Infant Development, *GCMS* gas chromatography mass spectrometry, *HPLC* high performance liquid-gas chromatography, *CDC* Center for Disease Control, *SNP* single-nucleotide polymorphism, *MDI* Mental Development Index, *PDI* Pervasive Development Index

**Table 6:** Neurobehavioural outcome explored in each

	Cognitive	Behavioural	Sensory	Motor	Morphology
Eskenazi et al. [5]	٭				
Eskenazi et al. [21]	٭	٭	٭		
Millenson et al. [26]	٭	٭	٭	٭	٭
D’Amelio et al. [27]		٭			
Pasca et al. [28]		٭			
Engel et al. [29]	٭			٭	

## Results

Based on the search strategy employed, six articles met the inclusion and exclusion criteria. Summary characteristics of the included studies appear in Table 1.

### Study design and location of the studies

In four of the articles included in this review, the studies followed a cohort study design [5, 21, 26, 29], whilst the remainder two articles followed a case-control study design [21, 28]. Two articles emanated from one study [5, 21]. Four of the papers reported on studies in the USA [12, 18, 21, 22]; one study involved Italian and Canadian participants [27] and one study was based in Romania [28]. Quality assessment of selected articles indicated that most of the articles selected for the study were of a good quality rating, except the article by Pasca et al. which did not report on pesticide exposure.

### Study participants and sample size

Four articles reported on mother and child pairs [5, 21, 26, 29], whilst one study reported on children [28]. The age of children assessed ranged from 1 to 9 years in the selected articles. One article assessed children at three stages (one, two and 6 to 9 years) [29], whilst another article assessed children at two stages (5 and 7 years) [21]. One article assessed children at only one age point, at 2 years [25] and one article at 8 years [26]. The age of children in one article was not reported [28].

### Exposure measurement

In three articles [5, 21, 29], urinary samples were taken to assess prenatal pesticide exposure. In one article [27], urinary peptides were measured. In all the articles, blood samples were taken for PON1 SNP genotyping and enzyme assessment. Four articles [5, 21, 26, 29] included in this review explored pesticide load using gas chromatography tandem mass spectrometry (GC/MS). One article [27] used high performance liquid chromatography (HPLC) [25] whilst another used an enzymatic activity assay [28] to quantify pesticide exposure.

### Genetic outcome analysis

Four articles reported on the *PON1* (108T) SNP [5, 21, 26, 27], whilst two articles reported on the *PON1* (L55M) SNP [27, 28]. All six articles reported on the *PON1* (Q192R) SNP. Other SNPs which have been explored by researchers were not included because of their associated health outcome being outside the scope of this study. It was deduced that various SNPs exhibit significant inter-ethnic differences, meaning their distribution differs among different ethnic and cultural groups [22].

### Neurobehavioural tools used to assess outcomes

Children assessed for neurobehavioural health outcomes were between the age(s) of 1 to 9 years old. Tools used to assess neurobehavioural health outcomes in the included studies were the Bayley Scale of Infant Development (BSID) (two articles [5, 29]) which measures the Mental Development Index (MDI) and Psychomotor Development Index (PDI), the Conners’ Kiddie Continuous Performance Test (K-CPT) (one article [21]), the Wechsler Intelligence Scale (WISC) for Children (one article [21]) and the Behaviour Assessment System for Children-2 (BASC2) (one article [26]). Two articles included in the review [27, 28] reported on autism spectrum disorder (ASD) and used a physician diagnosis of autism using the standard approved diagnostic method.

### Association of PON1 polymorphism with neurobehaviour health outcome in children (Tables 3 and 4)

Three articles reported a significant association between organophosphate exposure, PON1108T and Q192R polymorphisms, and neurobehaviour health outcomes [12, 18, 22]. No significant associations were observed in the remaining three articles [23–25]. Eskenazi et al. quantified maternal urine dialkyphosphate (DAP) levels, genotyped the PON1 gene for PON1 192 and PON1 108 polymorphism and measured the enzymatic activity of paraoxonase (POase) and arylesterase (ARYase). The PON1 108T allele in children was associated with poorer MDI scores and high DAP levels [5]. However, other PON1 polymorphic variants and enzymes showed no significant association. The association of the PON1 108TT allele with poorer/low neurofunction scores was further reported in a follow-up study of the same cohort [21]. In this paper [22], maternal PON1 108T allele carriers showed a weakly modified relationship between DAP and neurobehaviour. Engel et al. found the PON 1 Q192R QR/RR variant genotype to be associated with poorer perceptual reasoning and increasing dialkyl- and dimethylphosphate (DAP and DMP) concentrations in urine. The adverse neurobehavioural health outcome in children was observed from 12 months of age and continued up to 9 years of age [29]. The low enzymatic activity was associated with the PON1 Q192R allele and with exposure to DAP and DMP [21]. ARYase and POase levels and activity were also reported to be associated with the neurodevelopment hindrance, leading to autism spectrum disorders in Romanian children [28]. However, the above-mentioned study did not find any significant association in PON1 allelic distribution [28]. The PON1 R192 gene variant was implicated in the pathogenesis of autism among North American (Caucasian), but not Italian, organophosphate users [27].

## Discussion

The articles show that organophosphate (OP) and metabolite exposure is implicated in the pathogenesis of adverse neurobehavioural health outcomes. Pesticide application at home or in the work place may play a role in the development of adverse neurobehavioural health outcomes, especially if exposure is directed to pregnant women who are likely to transfer their pesticide load to the unborn child [21]. The pesticide load is likely to cause prominent changes on the genetic variability of selected genes, as the chemical structure of pesticides has been proven to be genotoxic and ultimately neurotoxic [6]. The articles included in this study show that pesticide exposure (in this case organophosphate and metabolites) induces a pressure on the PON1 gene, which resulted in low enzymatic activity of specific enzymes and has been associated with low neurobehavioural scores [5, 22, 28, 30].

Polymorphic variants on the *PON1* gene show great variability which may influence the activity and functionality of enzymes encoded by that gene [29]. The PON1 SNPs which have been implicated with adverse neurobehavioural function are the PON1 108T and Q192R [21, 29]. The PON1 L55M allele has been implicated in the aetiology of other health outcomes (i.e. atherosclerosis, cancer and leukaemia) besides those of neurobehaviour dysfunction [27]. Maternal PON1 can affect polymorphic status of neurobehaviour function in children [21]. The PON1 L55M allele is rather associated with influencing the plasma enzymatic levels [3]. Prenatal exposure to OP is likely to induce low enzymatic activity (ARYase and POase) which consequently leads to reduced neurobehavioural function scores [5]. The reduction in neurobehavioural function score can be observed from 1 to 9 years of age [5, 29]. The PON1 192R(+) allele was found to be common among pesticide exposed groups and was associated with increased paraoxonase and acetylcholinesterase activity [31].

Prenatal exposure to OP may place children at risk of developing neurobehavioural hindrance, in some cases leading to the development of autism and autism spectrum disorders (ASD) [27, 28]. In the two articles included in this review, the PON1 108T allele was associated with autism but not significantly [20, 21]. This non-significance may be because of the small sample size in the studies. The PON1 108T (+) and the 192R (+) alleles have been associated with adverse health outcomes in other studies [3, 31].

Neurobehavioural function and PON1 polymorphic status, due to OP exposure, may be difficult to grade as it is age and ethnic group dependant [21, 27]. However, the researchers in the articles used standardised age appropriate tests. What would have been interesting would be if all the studies had used similar neurobehavioural function screening tests and exposure screening tools, enabling a more in-depth analysis of the results of the studies included.

## Limitations of this review

This was a comprehensive review of the literature and was inclusive of all nationalities or geographical location literature, as long it was in line with the search term combination used. However, there are some limitations which need to be noted. Our search was limited to articles published between 2000 and 2018 with the result that articles published prior to this date that may contain important information have not been included in this review. English language articles were only considered, and so studies in other languages may have been omitted. The studies were largely in Caucasian populations, and studies in other ethnic groups may show different findings.

A further limitation is that not all the papers clearly defined the exact pesticide exposure concentration that is associated with an adverse health outcome. Articles not clearly reporting on the pre- and postnatal exposure to organophosphate and/or any of its metabolites could not be included in this review. Most articles in line with the study title were not included because of the pesticide of interest and health outcome. It was observed that most articles explored pesticide exposure in association with a variety of health outcomes which were not of interest to the current study. This would have affected the synthesis of this study differently as there are many studies which have evaluated the association of the PON1 gene with other health outcomes besides the neurobehavioural ones. There were articles, which explored the association between the PON1 SNP and neurobehavioural health outcomes, and other environmental toxicants besides organophosphates. Most articles used urine and blood samples to quantify pesticide load, whilst others used enzymatic activity.

## Conclusion

This systematic review of the peer reviewed literature shows an association between prenatal organophosphates exposure and PON1 SNP, with neurobehavioural health outcomes. According to our knowledge, this is the first systematic review which explored the association of pesticide exposure with PON1 genotype and neurobehavioural health outcomes. 

Association between prenatal pesticide exposure and SNP presence on PON1 gene needs to be further investigated to obtain a succinct understanding of how it can affect neurobehaviour. Current literature indicates that prenatal pesticide exposure affects neurodevelopment by influencing the genotype of the PON1 gene, which subsequently alters POase and ARYase activity.

## Data Availability

For this manuscript supporting data can be made available on request from the first author.

## Abbreviations

ARYase: Arylesterase
ASD: Autism spectrum disorder
ADHD: Attention deficit hyperactivity disorder
BSID: Bayley Scale of Infant Development
BASC2: Behaviour Assessment System for Children-2
DAP: Dialkylphosphate
POase: Paraoxonase
K-CPT: Conners Kiddie Continuous Performance Test
PDI: Psychomotor Development Index
MDI: Mental Development Index
PON1: Paraoxonase-1
SNP: Single nucleotide polymorphism
OP: Organophosphate
WISC: Wechsler Intelligence Scale
